# Retroperitoneoscopic resection of retroperitoneal nonadrenal ganglioneuromas: our technique and clinical outcomes

**DOI:** 10.1590/S1677-5538.IBJU.2017.0460

**Published:** 2018

**Authors:** Changjin Shi, Feng Li, Yanchao Wang, Long Pei, Tao Wang

**Affiliations:** 1 Department of Urology, the Fourth Affiliated Hospital of Hebei Medical University, Shijiazhuang, China; 2 Department of Nephrology, Hebei Provincial General Hospital, Shijiazhuang, China

**Keywords:** Retroperitoneal Neoplasms, Ganglioneuroma, Pathology

## Abstract

**Objective:**

To report our experience of retroperitoneoscopic technique in semi-lateral decubitus position for the retroperitoneal nonadrenal ganglioneuromas in 18 patients, and to evaluate its clinical outcomes.

**Materials and Methods:**

From January 2012 to May 2016, 18 patients with retroperitoneal nonadrenal ganglioneuromas underwent retroperitoneoscopic resection. With the patients in semi-lateral decubitus position, a 4-port retroperitoneal approach was used. Data were collected on the tumor size, tumor location, perioperative outcomes, pathology, and last-known disease status. We reviewed the operative videos to identify surgical tips and tricks.

**Results:**

All procedures were carried out successfully without converting to open surgery. The tumors had an average size of 5.2cm. The mean operative time was 86.5 min, with a mean estimated blood loss of 85.4mL. There were three patients suffering from intraoperative complications. Postoperatively, all patients achieved an uneventful recovery; the mean postoperative hospital stay was 5.5 days. The postoperative pathology revealed to be retroperitoneal ganglioneuromas. With a mean follow-up of 39.5 months, all patients were recurrence free. The review of the operative videos revealed several tips and tricks, including keeping peritoneum and posterior Gerota fascia intact to provide a favorable operative exposure of tumors, and placing the harmonic scalpel through different ports during tumor dissection.

**Conclusions:**

With the patient in semi-lateral decubitus position and a 4-port retroperitoneal approach, retroperitoneoscopic resection of retroperitoneal nonadrenal ganglioneuroma is a feasible, effective, and safe procedure. This approach has distinct advantages including direct access to the tumor, optimal exposure of tumor and less intraperitoneal interference.

## INTRODUCTION

Ganglioneuromas are rare, benign neurogenic tumors that originate from the neural crest. Arising along the sympathetic chain, ganglioneuromas are commonly localized in the posterior mediastinum followed by retroperitoneum, cervical region and adrenal gland (1). Retroperitoneal ganglioneuromas are usually nonfunctioning and asymptomatic until they reach large sizes, in which case they cause symptoms due to local expansion and pressure on adjacent structures (2). Surgical resection represents the only choice for both diagnosis and treatment (3).

Retroperitoneal tumors have traditionally been excised using a standard open technique (47). Recently, due to advances in laparoscopic instruments and surgical techniques, indications for the laparoscopic approach have been broadened to the retroperitoneal tumors (8-10). However, surgical access to the retroperitoneal space is generally achieved by abdominal transperitoneal approach. Reports on the retroperitoneal laparoscopic approach to nonadrenal retroperitoneal tumors are limited (11). In comparison with transperitoneal laparoscopic surgery, the main advantages of retroperitoneal approach include a faster access to the tumor, requiring little dissection without violating the peritoneal cavity. Herein, we report our experience of retroperitoneal laparoscopic resection of nonadrenal retroperitoneal ganglioneuroma in 18 patients and analyze the feasibility and safety of our technique.

## MATERIALS AND METHODS

From January 2012 to May 2016, 18 patients (6 males and 12 females) underwent retroperitoneoscopic resection of nonadrenal retroperitoneal ganglioneuromas. The average age of the patients was 40.6 years, ranging from 21 to 65 years. The therapy modality was approved by the hospital ethics committee and written informed consent from patients was obtained prior to surgery. Preoperative assessment of each patient's general condition was carefully performed, including routine blood laboratory investigation, coagulation profile, urinalysis, hepato-renal function. Laboratory data and the tumor markers (neuron-specific enolase, NSE, serum carcinoembryonic antigen, CEA and carbohydrate antigen 199, CA-199) were all within normal limits. The patient's catecholamine levels in 24-hour urine samples were measured to exclude paragangliomas. All patients were evaluated preoperatively with abdominal computed tomography (CT) or magnetic resonance imaging (MRI). The mean tumor size was 5.8cm the tumor location was suprahilar in 4, and infrahilar in 14 cases. The patient's characteristics and operative data are summarized in Table-1. Abdominal CT was performed 3 and 6 months postoperatively. Thereafter, follow-up was then continued at 6-month intervals.

**Figure 1:**
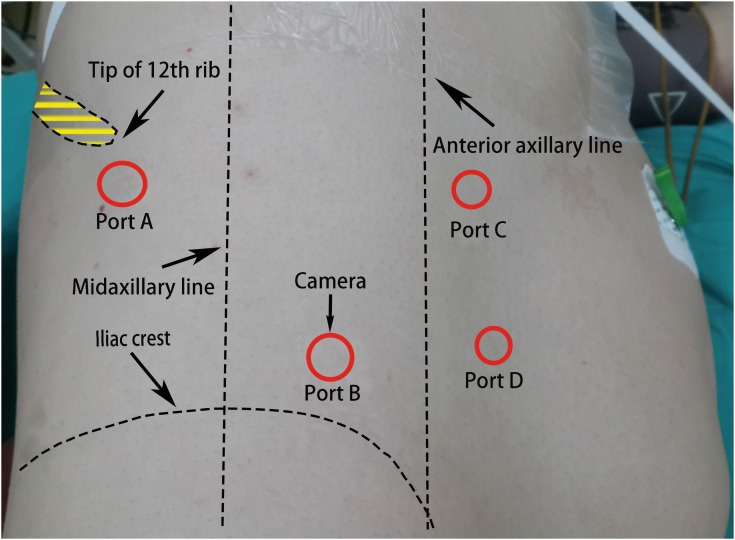
Patient position and distribution of trocars. The patient was placed in the semilateral decubitus position with the side of the lesion elevated at 60°. Trocar A, below the tip of 12th rib. Trocar B, 2-cm above the superior border of the iliac crest and medial to the midaxillary line. The other two trocars (C and D), along the anterior axillary line and 2-3 cm towards the midline.

After induction of general anesthesia, the patient was placed in the semi-lateral decubitus position with the side of the lesion elevated at 60. A 2cm skin incision was made below the tip of 12th rib (point A) (Figure-1). The retroperitoneal space was entered using sharp and blunt dissection through the flank abdominal muscles and lumbodorsal fascia, and then an index finger was inserted for a simple dissection to develop an initial retroperitoneal pocket. A homemade latex balloon dissector was placed into the retroperitoneal space, and 800-1000mL air was infused to maintain the balloon dilation for 3-5 minutes. The air was then evacuated, and the balloon dissector was removed. Under the guidance of the index finger extending into the retroperitoneal space through the incision, a 10-mm trocar was inserted 2cm above the superior border of the iliac crest and medial to the midaxillary line (point B), the other two 5-mm trocars were placed along the anterior axillary line and moved 2-3cm toward the midline (point C and D). A 10mm trocar was inserted at point A, and the skin incision was closed around the port using a mattress suture to avoid gas leakage. The laparoscope was placed through the trocar at point B, which was connected to the carbon dioxide insufflator to achieve the pneumoretroperitoneum (pressure range, 13-15mm Hg). The retroperitoneal fat was partially freed to reveal the lateral conal fascia, which was then incised longitudinally. Dissection proceeded over the quadratus lumborum and then to the psoas muscle. Tumor was easily identified in the retroperitoneal space adjacent to the medial of the psoas muscle, and it was dissected and mobilized from adjacent structures. In order to facilitate the manipulation of the tumor, the harmonic scalpel was placed through ports C for dissection of the upper pole of the tumor, port D was used to retract. For dissection of the lower pole of the tumor, the harmonic scalpel was switched to port D, port C was used to retract. Hemostasis was checked carefully after lowering the pressure of the pneumoretroperitoneum. A closed suction drain was placed through the port B into the space. Carbon dioxide was evacuated, and the port sites were closed. The closed suction drain was subsequently removed if the drainage output had not increased and was less than 10mL in 24 hours.

**Table 1:** Patients’ characteristics and surgical outcomes.

Patient No./Sex/ Age (y)	Symptom	Tumor location	Tumor size (cm)	Operative time (min)	Blood loss (mL)	Intraop/postop complications	Postop stay (days)	Follow-up time (months)
1/F/46	No	Left/suprahilar	5.8	121	77	No	6	64
2/M/40	No	Left/infrahilar	4.8	82	80	No	4	58
3/F/34	No	Right/infrahilar	3.2	113	86	peritoneum breach	5	54
4/M/33	Abdominal pain	Left/infrahilar	6.4	97	90	No	5	52
5/F/55	No	Right/infrahilar	7.1	62	60	No	6	52
6/F38	Abdominal pain	Right/infrahilar	6.6	72	98	No	7	50
7/F/65	No	Right/suprahilar	4.7	105	70	peritoneum breach	5	46
8/M/46	No	Right/infrahilar	5.1	68	50	No	4	45
9/F/52	Left flank pain	Left/suprahilar	6.2	84	75	No	5	42
10/M/29	No	Right/infrahilar	7.4	102	260	lumbar vein injury	7	39
11/M/62	Abdominal pain	Right/infrahilar	5.8	75	60	No	6	38
12/F/27	No	Right/infrahilar	3.6	69	50	No	4	35
13/F/34	No	Right/infrahilar	4.8	78	58	No	5	33
14/F/21	No	Right/infrahilar	7.8	89	90	Chylous leakage	9	29
15/F/54	Right flank pain	Right/infrahilar	6.3	93	65	No	6	24
16/M/39	No	Left/infrahilar	4.8	69	80	No	4	20
17/F/25	No	Left/infrahilar	6.3	72	78	No	5	18
18/F/31	Abdominal pain	Right/suprahilar	8.2	106	110	No	6	12

## RESULTS

**Figure 2:**
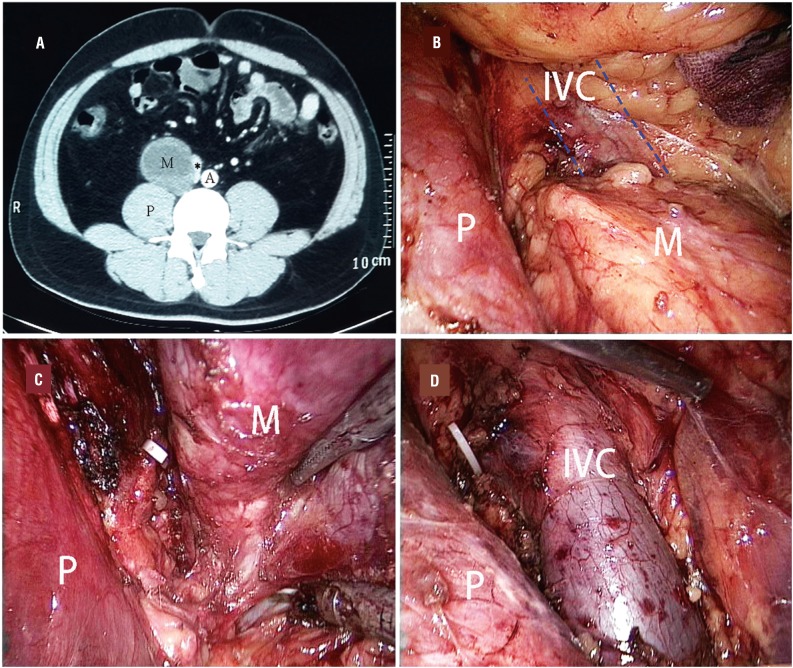
Preoperative CT and intraoperative findings of patient 6. A) Preoperative CT scan image showing a large tumor located between the IVC (asterisk) and the psoas muscle. The IVC was compressed medially. B) The tumor was identified after entering the retroperitoneal space. It was located medially to the psoas muscle. The IVC was compressed medially to the tumor. C) Laparoscopic view during dissection. The IVC had been released from the media side of the tumor. D) Laparoscopic view after resecting the tumor. CT indicates computed tomography; A = aorta; IVC = inferior vena cava; M = mass; P = psoas muscle.

The detailed characteristics and perioperative data of the patients are summarized in Table-1. Of the 18 treated patients, 12 patients were asymptomatic and detected incidentally during health screening. All operations were completed laparoscopically without conversion to open surgery. Intraoperatively, the tumors appeared to be well-encapsulated and were mostly dissected free from adjacent structures easily (Figure-2). Surgical time ranged from 62 to 121 minutes, with an average of 86.5 minutes. The mean blood loss during the operation was 85.4mL (range, 50-260mL), and none of the patients required blood transfusion. There were three patients suffering from intraoperative complications, one with the lumbar vein injury and other two with the peritoneum breach. During the operations, there were no instances of ureter and renal pedicle injury in these cases. Regarding the postoperative complications, chylous leakage was observed in one patient and was managed conservatively. All other patients achieved an uneventful recovery. Oral intake was resumed after a delay of 2 days (range, 1-3 days) after surgery. The mean postoperative hospital stay was 5.5 days (range, 4-9 days). Postoperatively, histopathologic examination results revealed ganglioneuroma in all the patients. All resected specimens showed a negative incisional margin. At a mean follow-up of 39.5 months (range, 12-64 months), abdominal computed tomography showed no recurrence in all patients.

We reviewed our surgical videos and clarified several technical tips and tricks. Keeping peritoneum and posterior Gerota fascia intact were helpful to obtain a favorable operative view. In 2 cases with the peritoneum breach, a Veress needle was placed in the umbilicus, but it didn't fully resolve the impingement of the retroperitoneal working space. In the cases with tumors located above the renal hilum, rotating the kidney was helpful to approach the tumors. It was helpful for facilitating the tumor manipulation to place the harmonic scalpel through different ports alternately during tumor dissection, especially in the cases with tumors located below the renal hilum.

## DISCUSSION

Ganglioneuromas are tumors of the sympathetic nervous system that arise from the neural crest cells (12). They are accepted as slow-growing benign tumors constituted by mature sympathetic ganglion cells (13). Whereas ganglioneuromas can be found everywhere along the sympathetic chain, the posterior mediastinum, retroperitoneal area, and adrenal glands are the most common locations. Ganglioneuromas primarily affect the pediatric age group, two-thirds of patients are under the age of 20 years, and ganglioneuromas are rarely observed over the age of 60 years (14). They are mostly sporadic but there are a few reports of ganglioneuromas associated with neurofibromatosis type II and multiple endocrine neoplasia type II B (15). They are common in young females and usually asymptomatic until they reach a large size when they compress and displace adjacent structures (6, 16). Ganglioneuromas rarely produce vasoactive intestinal polypeptide and catecholamines. These tumors may cause some symptoms like diarrhea, sweating and hypertension related to those peptides (17). In our cases, the patients presented with a retroperitoneal mass that did not have secretory activity.

The current advanced imaging techniques may be useful for evaluating the extent of the ganglioneuromas and differential diagnosis. CT most commonly reveals a homogenous and well-encapsulated tumor with non-enhancement or slight enhancement in arterial phase and progressive mild enhancement in delayed phase. Circumscribed or spotted calcification may be observed in 20% of the patients (18). On MRI, T1-weighted images show a low-signal intensity, whereas T2-weighted images show a heterogeneous high-signal intensity (14, 19). Fine-needle aspiration (FNA) can be used preoperatively, but it usually leads to inconclusive diagnosis. In the largest series with ganglioneuromas of presacral location, the diagnosis could not be achieved in 60% of cases with FNA (20). In particular, although a catecholaminergic crisis has never been described subsequent to FNA, this theoretical possibility exists (21). Without intention to perform FNA due to inconclusive results and the possibility of catecholaminergic crisis, we considered a benign neurogenic tumor as the presumed diagnosis according to CT and MRI features and lack of enhancement of the lesion. CT and MR imaging can demonstrate important characteristics of these tumors and help narrow the differential diagnosis; however, there is a substantial overlap of imaging findings among different tumors. We have 4 cases of misdiagnosis in our experience. Based on the CT characteristics, they were diagnosed as ganglioneuromas before surgical resection, whereas the postoperative pathology revealed schwannomas which were not included in this article. Here, we only selected patients whose postoperative histopathologic examination revealed ganglioneuromas.

Retroperitoneal tumors were excised traditionally by laparotomy (4-7). However, in recent decades, with advances in laparoscopic technique and the associated equipment, laparoscopic excision for some retroperitoneal tumors is the ideal approach nowadays (8-10). The laparoscopic approach has been associated with fewer postoperative complications including less blood loss, minor postoperative adhesion formation, and shorter hospital stay than laparotomy. Laparoscopic retroperitoneal tumor excision can be performed through the retroperitoneal or transperitoneal approach. However, surgical access to the retroperitoneal space is generally achieved by abdominal transperitoneal approach. Reports on the retroperitoneal laparoscopic approach to nonadrenal retroperitoneal tumors are limited. In comparison with transperitoneal laparoscopic surgery, the main advantages of retroperitoneal approach include a faster accessing to the tumor, requiring little dissection without violating the peritoneal cavity. Walz et al. previously reported their experiences of laparoscopic or retroperitoneoscopic surgery for 27 paragangliomas. They used the prone position combined with a gas pressure of 20-24mmHg in retroperitoneoscopic surgery (22). Zhang S et al. reported their retroperitoneoscopic technique in supine position for the primary tumors located below the level of renal pedicle (23). In our surgical technique, we also preferred the retroperitoneal approach on the basis of our extensive experiences. But, we used semi-lateral decubitus position.

The incidence of retroperitoneal tumors is too infrequent for most surgeons to gain sufficient experience in laparoscopic excision. In our cases, we selected the tumors located below or above the level of the renal pedicle. Therefore, for an experienced surgeon who is adroit at retroperitoneoscopic adrenalectomies, nephrectomies and others, retroperitoneoscopic resection of a retroperitoneal tumor below or above the level of the renal pedicle can be performed easily. Since the tumor was partially sheltered from the psoas muscle, we modified the patient position as used in nephrectomy. We performed the procedure with the patient in a semi-lateral decubitus position in order to get an optimal exposure of the tumor. We slightly modified port positioning which we used in retroperitoneoscopic nephrectomy. Briefly, all four ports were moved 2-3cm toward the midline in order to facilitate the exposure and manipulation of the tumor.

In our experience, dissecting along the surface of the psoas muscle was sufficient to expose the tumor. The posterior Gerota fascia and perinephric fat should be left intact to keep its adherence to the peritoneum, which can play a role of “self-retraction” to avoid dropping of the fascia, otherwise may prevent the surgeon's ability to maneuver. Maintaining the integrity of the peritoneum is a key factor during the retroperitoneal performance. However, the peritoneum could be damaged and opened inadvertently, losing the surgical field exposure advantage provided by the pneumoretroperitoneum. There were two cases suffered from the peritoneal breach in our study. They occurred during the trocar placement. Extended and more careful finger dissection to separate the adherent peritoneum from the abdominal wall may reduce this complication. Furthermore, incising the lateral conal fascia longitudinally along the quadratus lumborum, which is far away from lateral peritoneal reflection, may further contribute to preventing peritoneal injury.

Arising along the sympathetic chain, retroperitoneal ganglioneuromas are commonly located in a deep, narrow space and adjacent to major vessels, so it is difficult to perform a laparoscopic resection of the tumors especially when tumors are adherent to adjacent major vessels. However, retroperitoneal approach affords rapid and direct access to the tumors, with the retraction of psoas muscle and Gerota fascia, the laparoscopic magnification provides an excellent exposure. In our cases, all operations were completed laparoscopically without conversion to open surgery. When a tumor adhered to important adjacent vessels, as showed in our patient 6, the tumor adhered to the inferior vena cava, meticulous dissection was necessary. During the tumor dissection, port C and D could offer different operative direction around the tumor. As a surgical tip, we found it was helpful for facilitating the tumor manipulation to place the harmonic scalpel through ports D and C, alternately.

Because of the benign nature of ganglioneuromas, adjuvant systemic chemotherapy or local radiotherapy are not indicated after surgical resection. As ganglioneuromas have a tendency to remain silent for a long time, and are often associated with a long-term disease-free survival (6), regular follow-up is necessary to assess local recurrence. In our patients, recurrence has not been observed at a mean follow-up of 39.5 months.

We present a small retrospective study, more cases and further follow-up are still needed to establish that retroperitoneoscopic resection does not have a deleterious effect on the longterm outcome. Secondly, our study could not answer the question of whether laparoscopic surgery is a viable option for malignant retroperitoneal tumors. However, we consider that our study further supports the feasibility of retroperitoneal laparoscopic resection of retroperitoneal ganglioneuromas in experienced hands, and we offer several surgical tips and tricks.

## ABBREVIATIONS

NSE: = neuron-specific enolase
CEA: = serum carcinoembryonic antigen
CA-199: = carbohydrate antigen 199
CT: = computed tomography
MRI: = magnetic resonance imaging
FNA: = fine-needle aspiration
